# Emetin: A Note of Warning

**Published:** 1917-02

**Authors:** 


### EMETIN: A NOTE OF WARNING.

While it may be accepted as a truism that remedies marketed under fancy proprietary names too often are not what they pretend to be and do not accomplish what they claim, it must not be lightly assumed that the converse is equally true. The physician is rightly suspicious of such products; but on the other hand he should not take it for granted that because a drug bears the name of a definite chemical compound, it is true to name and pure, and therefore trustworthy in its actions. Of this fact we have recent demonstration in respect to emetin. The use of this drug has greatly increased, during the last two or three years, owing to its extensive employment in pyorrhea alveolaris and in amebic dysentery. Furthermore, it is being used by certain mail-order "pyorrhea cure" quacks. It is administered in quantities not far removed from the subtoxic dose, while, from the very nature of the maladies in which

* Levy, R. L., and Rowntree, L. G.: On the Toxicity of Various Commercial Preparations of Emetin Hydrochlorid, Arch. Int. Med., March, 1916, p. 420.

it is used, the patients are frequently in a condition of exhaustion. These facts combine to render it vitally important that, in prescribing this remedy, we should be assured that it is of uniform composition and pure. Nor, in this particular instance, is there any reason why this standard should not be constantly attained.

For this reason, a recent article in the *Archives of Internal Medicine** must have come as a rude awakening to those who, relying on the conscientiousness of the manufacturer, and the reputed harmlessness of emetin, have been employing this potent remedy with an easy mind.

Two cases from the Johns Hopkins medical clinic are described in which symptoms of poisoning and in one instance death resulted from the administration of emetin hydrochlorid. The fatal case occurred in a man who entered the hospital with a diagnosis of syphilis and amebic dysentery. He was treated for twenty days by subcutaneous injections of emetin hydrochlorid. The average daily dose was 1½ grains; the total amount he received was 29 grains. A pre-existing slight diarrhea was at first ameliorated and then markedly intensified till, on the eighteenth day of the treatment, there were eighteen stools in the twenty-four hours. This diarrhea ceased five days after the discontinuance of the injections. From the sixteenth day on, signs of grave kidney mischief developed. There were marked acidosis and acute renal insufficiency, with blood in the urine. Bronchopneumonia supervened and, after a period of dyspnea, the patient collapsed and died thirty days after the first injection and ten days after the last.

The other patient was a woman who received only one-half grain daily during four days, for pyorrhea alveolaris. She developed intense diarrhea with pain and tenesmus. These symptoms cleared up six days after withdrawal of the emetin. The patient was then in a toxic delirious state which lasted one week. These symptoms were quite out of proportion to the moderate dose employed. The particular preparation employed in this second case was, therefore, suspected to be unusually toxic. This suspicion was confirmed by an elaborate experimental research into the toxicity of emetin hydrochlorid preparations obtained from five different commercial sources, in the course of which investigation sixty-two animals were used. The toxic effects manifest themselves in various ways, but in using emetin one must be on the lookout for such danger signals as intense diarrhea, albuminuria, and peripheral neuritis.

It is true that this fatal case is the first recorded in the literature, while instances of grave toxic effects have not often been reported. But these facts must not be accepted at their face value. Two peculiarities of the action of emetin (or of the associated impurities) conspire to mislead. In the first place, certain of the toxic effects closely imitate the symptoms of dysentery, the very disease in which emetin is most extensively used in relatively large doses. Secondly, the maximum poisonous effect tends to be deferred, occasionally manifesting itself some days after the drug has been discontinued. The phenomena of intoxication are, therefore, apt to be interpreted as an exacerbation of the pre-existing morbid condition.

The case of emetin is not unique in the history of pharmacology. At one time the products marketed under the name of ''aconitin'' varied startlingly in their toxicity. At that period the chemistry of aconitin and of its congeners had been very inadequately investigated. No such excuse avails regarding impure emetin. This alkaloid is a well defined chemical compound. It is commercially practicable to ascertain, with precision, the emetin content of the hydrochlorid and to insure its freedom from dangerous impurities. In the article referred to it is proved, clinically and experimentally, that at least one house of repute is supplying, under the name of emetin hydrochlorid, a product so unusually toxic as to prohibit its use, at any rate in anything approaching the ordinary dose.

It is open to the manufacturer referred to to say that its greater toxicity is due to its greater purity. There is, however, nothing in the facts which supports such a contention, and there is much which militates against it. However this may be, one indisputable fact stands out: The products supplied as emetin hydrochlorid are variable in composition and in toxicity to a degree which constitutes a serious danger. It therefore behooves physicians to insist on some declaration from the firm supplying emetin hydrochlorid as to its purity and as to the standard employed.—Editorial in *Journal A. M. A.*, April 22, 1916.

